# The Story behind the Science: On the discovery of respiratory syncytial virus

**DOI:** 10.1128/mbio.03074-24

**Published:** 2025-01-21

**Authors:** Arturo Casadevall, Philip R. Roane, Thomas Shenk, Bernard Roizman

**Affiliations:** 1 Department of Molecular Microbiology and Immunology, Johns Hopkins Bloomberg School of Public Health, Baltimore, Maryland, USA; 2 Department of Microbiology, Howard University College of Medicine, Washington, DC, USA; 3 Department of Molecular Biology, Princeton University, Princeton, New Jersey, USA; 4 Cummings Life Sciences Center, The University of Chicago, Chicago, Illinois, USA; Columbia University Medical Center, New York, New York, USA

**Keywords:** RSV, respiratory viruses, discovery

## Abstract

Respiratory syncytial virus (RSV) was discovered in 1956 by the laboratory of Robert Chanock after its isolation from children with upper respiratory infections. Here, we review the events leading to its discovery including its prior isolation as chimpanzee coryza virus and its subsequent association with human disease.

## COMMENTARY

Today, respiratory syncytial virus (RSV) is the leading cause of infant hospitalizations (1) and a significant cause of morbidity and mortality among older individuals (2). On 3 May 2024, the Johns Hopkins Bloomberg School of Public Health was designated a “Milestones in Microbiology” site for discoveries related to microbiology, which included the discovery of RSV in 1957 by the collaborating laboratories of Robert Chanock and Bernard Roizman (3). Here, we revisit that seminal work with one of the authors of the original paper (B.R.) and review details of the early experimental work, the participants. and the events leading to the discovery of RSV.

To understand the time of RSV discovery in late 1950s, it is important to revisit virology at the mid-20th century. In 1949, Enders et al. succeeded in growing poliovirus in laboratory cell cultures for the first time, thus creating the conditions for the generation of vaccines for the prevention of poliomyelitis (4). This discovery, together with the introduction of continuous cell culture lines, made the 1950s a golden time for virological research. That decade saw the discovery of such human pathogenic viruses as varicella zoster virus, measles virus, and rhinoviruses. Work with bacterial viruses (bacteriophages) led Hershey and Chase to firmly establish that DNA and not protein carried genetic information (5), confirming earlier work implying that DNA was the transforming principle in pneumococcal transformation (6). The year 1957 saw the discovery of interferon (7), which would lead to fundamental discoveries in cell biology, immunology, and the mechanisms by which viruses both trigger and undermine cellular defenses (8). Capping this remarkable decade, Temin and Rubin showed that Rous sarcoma virus could transform tissue culture cells (9), launching (and supercharging!) the field of viral tumorigenesis.

**Fig 1:**
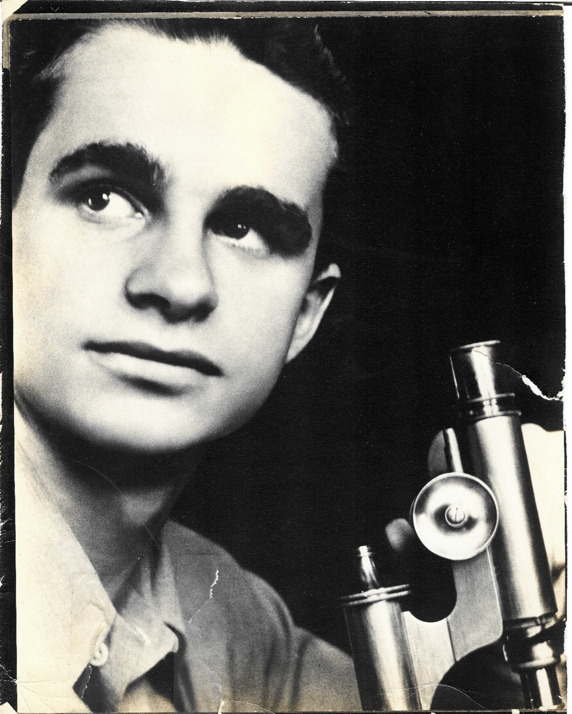
Dr. Robert Chanock as he appeared in the late 1950s. Photo courtesy of his son Stephen Chanock.

The story of RSV began with the outbreak of respiratory illness among chimpanzees at Walter Reed Army Institute of Medical Research (10). A virus was recovered from an infected chimpanzee with coryza that was propagated in epithelial-like cells derived from a human liver and shown to be transmissible by experimental infection of other chimpanzees (10). The investigators named the new virus as chimpanzee coryza agent (CCA). A laboratory worker in the research group, who worked intimately with the chimpanzees, developed an upper respiratory illness characterized by nasal stuffiness, runny nose, malaise, cough, and several days of fever. This individual made a specific antibody response to CCA (10). Barrack mates of this individual also had positive serology for CCA, suggesting possible human-to-human spread. The research team tested the human serum for complement fixing antibodies to CCA and found that it was present in about 20% of adolescent individuals. Hence, Morris, Blount, and Savage at the Virus Department at Walter Reed described a new virus from chimpanzees that was transmissible to humans and found serological evidence for its circulation in human populations. The chimpanzee CCA virus would eventually become RSV, but that chapter of the discovery would take place in the laboratories of Robert Chanock (Fig. 1) and Bernard Roizman in the Johns Hopkins School of Public Health.

**Fig 2:**
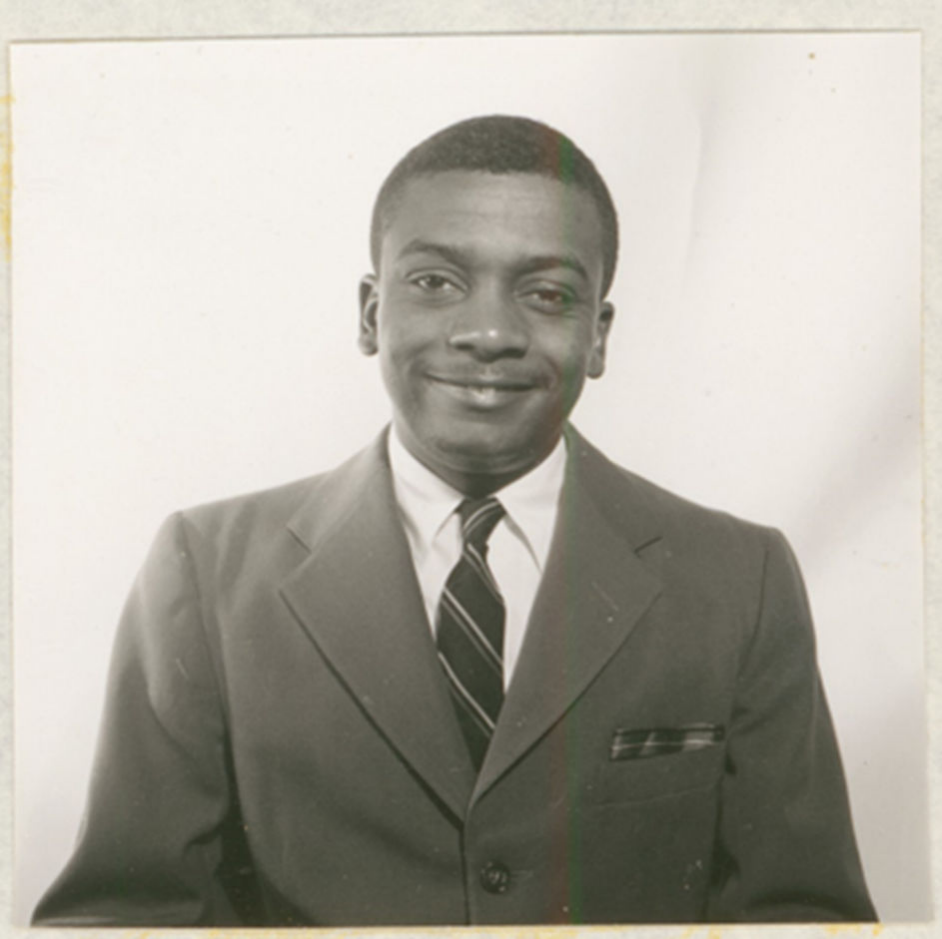
Dr. Philip Roane as he appeared in 1958. He received a master’s degree from the Department of Microbiology and Immunology of the Johns Hopkins School of Public Health in 1960. The photo is from the 1958 student directory photo, courtesy of the Alan Mason Chesney Medical Archives of the Johns Hopkins Medical Institutions.

Before moving to the National Institutes of Health in 1957, Chanock, working in the Departments of Epidemiology of the Johns Hopkins School of Public Health (then known as the Johns Hopkins School of Hygiene and Public Health) and Pediatrics of the Johns Hopkins School of Medicine, had a research laboratory near the Roizman laboratory in the School of Hygiene and Public Health. While at Hopkins, Chanock and Roizman together with Ruth Myers published a key paper on the discovery of RSV (3). Ruth Myers was a graduate of the Johns Hopkins School of Hygiene and Public Health. Their groups isolated two viruses known as “Long” and “Snyder” from children with lower respiratory illness (pneumonia) and showed that they were antigenically indistinguishable from the chimpanzee virus CCA. In collaboration with the Roizman laboratory, then at the adjoining Johns Hopkins School of Hygiene and Public Health, they carried out the first physical characterization of the virus showing that it consisted of particles with diameters of 90–130 µM purified using sucrose sedimentation with the assistance of Philip Roane (Fig. 2). Tissue culture studies established that the virus caused a cytopathic effect on KB cells (human epidermoid carcinoma cell line) in culture characterized by syncytial cytopathic changes. Furthermore, they established that this new virus was not related to adenovirus and different from other viruses known to trigger syncytial changes, such as mumps and measles viruses. In a second paper published back-to-back with the virological characterization of the “Long” and “Snyder” viruses in 1957, named after the patients from whom they were isolated, Chanock worked with Laurence Finberg, also at Johns Hopkins, to establish a firmer association among the Long, Snyder, and chimpanzee CCV viruses with human disease. They reported that 80% of healthy children had neutralizing antibody to “Long virus” by the age of 4 (11). Although these early epidemiological studies were not sufficient to establish causality, there was sufficient evidence for the “Long” and “Snyder” viruses to be defined as new agents of human disease. Their ability to form syncytia led Chanock and Finberg to propose grouping them with CCA under the name “respiratory syncytial” or “RS”, which became RSV (11) when coupled with the word virus. Subsequently, an epidemic of lower respiratory infection in Chicago in 1958–1959 was associated with a virus that was very similar to CCV and the “Long” and “Snyder” viruses, which was named Randall virus (12), which presumably was RSV, but that name was not adopted. Parenthetically, some online sources credit Maurice Hilleman with the discovery of RSV (https://www.utmb.edu/pedi/news/news-article-page/2023/09/26/rsv-awareness), but in an authoritative review written in 1963 Hilleman clearly credits the Walter Reed and Hopkins groups with the discovery of this virus (13).

A key aspect of the discovery of RSV was the purification of viral particles. In the late 1950s, the cutting-edge technology for virus purification involved density centrifugation, but there were very few virus laboratories with this expertise. Fortunately, the Roizman laboratory at Johns Hopkins had both the necessary equipment and expertise, and this led Dr. Chanock to seek their help. At the time, Dr. Philip Roane was a technician with Dr. Roizman and was an expert on density centrifugation, Mr. Roane subsequently obtained a Master of Science degree from the Johns Hopkins School of Public Health (class of 1960) and a PhD from the University of Maryland College Park (Fig. 2). Dr. Roane recalls that Dr. Chanock referred to the viral isolates as the “Long” virus and that he visited often overseeing the work. When Dr. Chanock saw the results of the gradients, he was so pleased that he gave him a bottle of scotch and a box of gourmet chocolates. Bernard Roizman and Philip Roane subsequently coauthored a series of foundational papers on polio virus, polyoma virus, and herpes simplex virus.

In reviewing the events associated with the discovery of RSV, it is striking that RSV was isolated almost simultaneously from chimpanzees and humans. Walter Reed and Johns Hopkins are separated by approximately 30 miles and the discovery of CCA and the “Long” and “Snyder” viruses that would all eventually become RSV occurred in 1955 to 1957. The phenomenon of temporally related discovery by independent groups is common in science and virology. For example, the discovery of reverse transcriptase by Temin and Baltimore occurred independently and nearly simultaneously (14). For RSV, the isolation from chimpanzees at Walter Reed and from infants at Hopkins undoubtedly reflects circulation of the virus in the region and that both institutions had state of the art virology facilities at the time. Given that the chimpanzee colony was in a research environment where the animals are maintained in a controlled environment, a plausible origin for primate CCA was the result of inadvertent infection of these animals by RSV carried by human caretakers.

Before the isolation of RSV, most cases of lower respiratory infections in children were attributed to influenza and parainfluenza viruses. The discovery of RSV provided a target for vaccine development. Unfortunately, unlike the discovery of poliovirus that led to effective vaccines in less than a decade, the road for RSV would be much more difficult. In the 1960s, the fateful formalin-inactivated RSV vaccine that elicited antibody responses was introduced but was ineffective and enhanced disease in vaccinees relative to those not vaccinated following infection with circulating RSV, such that 80% of vaccinees required hospitalization at the time of subsequent infection (15). This vaccine probably failed because the formalin-inactivated virus failed to trigger Toll receptor stimulation and subsequent affinity maturation to generate effective antibodies (16). It would take decades of work to unravel the structure of the virus and understand the basis of effective antibody immunity, knowledge that would eventually lead the Food and Drug Administration to approve the first vaccine against RSV in 2023. However, much remains to be discovered about the immunology and pathogenesis of RSV.

Today, RSV is a major pathogen for both the young and the old despite the availability of an effective vaccine and mAbs for prophylaxis. Antiviral therapy for RSV remains unsatisfactory, but ribavirin has found clinical utility in some populations. In 2001, human metapneumovirus was discovered, which is close a relative to RSV (17). Hence, the struggle against this virus and its close relatives continues but all the progress we have made dates to the discovery of the virus in the 1950s, which allowed the causal association with pneumonia and the eventual development of vaccines and antibody therapies for the prevention of disease.
